# Identification of novel drug targets for diamond-blackfan anemia based on *RPS19* gene mutation using protein-protein interaction network

**DOI:** 10.1186/s12918-018-0563-0

**Published:** 2018-04-24

**Authors:** Abbas Khan, Arif Ali, Muhammad Junaid, Chang Liu, Aman Chandra Kaushik, William C. S. Cho, Dong-Qing Wei

**Affiliations:** 1 Department of Bioinformatics and Biostatistics, College of Life Sciences and Biotechnology, Shanghai Jiao Tong University, Shanghai, 200240 China; 2 Department of Clinical Oncology, Queen Elizabeth Hospital, Kowloon, Hong Kong

**Keywords:** Diamond-Blackfan anemia (DBA), Microarray, Differentially expressed gene, KEGG pathways, Gene expression omnibus

## Abstract

**Background:**

Diamond-Blackfan anemia (DBA) is a congenital erythroid aplasia that usually presents in infancy. In order to explore the molecular mechanisms of wild and mutated samples from DBA patients were exposed to bioinformatics investigation. Biological network of differentially expressed genes was constructed. This study aimed to identify novel therapeutic signatures in DBA and uncovered their mechanisms. The gene expression dataset of GSE14335 was used, which consists of 6 normal and 4 diseased cases. The gene ontology (GO), as well as Kyoto Encyclopaedia of Genes and Genomes (KEGG) pathway enrichment analyses were performed, and then protein–protein interaction (PPI) network of the identified differentially expressed genes (DEGs) was constructed by Cytoscape software.

**Results:**

A total of 607 DEGs were identified in DBA, including 433 upregulated genes and 174 downregulated genes. GO analysis results showed that upregulated DEGs were significantly enriched in biological processes, negative regulation of transcription from RNA polymerase II promoter, chemotaxis, inflammatory response, immune response, positive regulation of cell proliferation, negative regulation of cell proliferation, response to mechanical stimulus, positive regulation of cell migration, response to lipopolysaccharide, and defence response. KEGG pathway analysis revealed the TNF signalling pathway, Osteoclast differentiation, Chemokine signalling pathway, Cytokine -cytokine receptor interaction, Rheumatoid arthritis, Biosynthesis of amino acids, Biosynthesis of antibiotics and Glycine, serine and threonine metabolism. The top 10 hub genes, AKT1, IL6, NFKB1, STAT3, STAT1, RAC1, EGR1, IL8, RELA, RAC3, mTOR and CCR2 were identified from the PPI network and sub-networks.

**Conclusion:**

The present study flagged that the identified DEGs and hub genes enrich our understanding of the molecular mechanisms underlying the development of DBA, and might shine some lights on identifying molecular targets and diagnostic biomarkers for DBA.

**Electronic supplementary material:**

The online version of this article (10.1186/s12918-018-0563-0) contains supplementary material, which is available to authorized users.

## Background

Diamond-Blackfan anemia (DBA) is a rare disease mostly affecting child during infancy. This disease is characterized by defect in red blood cells which result in the reduction or absence of erythroid precursors in bone marrow. The cephalic area is affected in most of the cases but other parts such as heart, limb and tract were also reported. This disease is a complicated disorder, however, its evolution remains erratic [1, 2]. Diamond-Blackfan anemia (DBA) is categorized as a rare genetic diseases characterized by cancer predisposition, bone marrow failure, pro-apoptotic haematopoiesis and congenital anomalies. It is also known as the inherited bone marrow failure syndromes (IBMFS) [3–5]. A wide range of mutations have been identified in DBA patients, from missense to nonsense mutations and from partial to complete deletion of one allele. DBA is the only disease known to be caused by defect in ribosomal protein [1, 2, 6, 7]. To date, DBA was reported as sporadic but latest cases approximately 45% of cases are familial [8]. DBA can be caused by mutations in the RPL5, RPL11, RPL35A, RPS7, RPS10, RPS17, RPS19, RPS24, and RPS26 genes [9]. However, RPS19 mutation account for 25% of the cases. RPS19 is one of the important proteins which interact with 18S rRNA and form the ribosomal small subunit 40S and perform its normal functioning of translation [1, 6]. Other than protein synthesis, RPS19 is also involved in many functions such as attraction of monocytes. Loss of non-ribosomal function could be the possible cause of DBA [10]. There is a case control study reported that patient with DBA has reduced RPS gene expression and so ribosomal synthesis defect is the underlying cause of DBA [11]. Some missense mutations in RPS19 was also reported to be involved in the reduction in the intracellular transport and stability of RPS19. With such mutations in cells are also reported to reduce differentiation and proliferation of erythroleukemic cell lines or CD34+ [12–14]. Furthermore, Chiocchetti et al. reported that RPS19 interact with PIM-1 oncoprotein in DBA. RPS19 with FGF-2 and with some other proteins of unknown function named S19-BP are also reported to interact with each others [15–17]. To date no definitive treatment is available for this complicated disease. Steroid therapy is widely used for the treatment which is responsive in 60% of the patients but sometimes patients suffered from severe complications such as iron overdose. DBA patients with steroid resistance required blood transfusion for a life time. Some cases successfully treated with interleukin-3 supplement [18], bone marrow transplantation [19] but not easy and affordable. It is necessary to find a feasible and cost-effective way of treatment.

Network based gene expression profiling is a proposed methodology to discover therapeutic signatures by integrating multiple factors including disease genes, gene expression intensities and proteins network [20–22]. Systems biology is one among such approaches, which rely on a global approach by analyzing the whole interacting network rather than a single protein, gene or enzyme to be analyzed. Systems biology reported that cellular protein does not function alone but these genes/proteins are clustered together to form an interconnected molecular networks to perform a specific function. Information regarding the individual gene/protein and function of a living system can be obtained through traditional approaches but systems biology alternatively access the mechanism at a systemic level. For this reason, systems biology employ biological relationships to construct a network consist of nodes (protein, enzyme, genes) interacting with another partner [23–26].

Proteomics and transcriptomic modeling of molecular networks from microarray data to discover potential biomarkers in DBA has not yet been resolved. Therefore, we used gene expression data by using computational systems biology approach based on microarray dataset analysis to identify the possible therapeutic gene/protein signatures for the treatment of DBA. Initially statistical approaches were applied to identify differentially expressed genes (DEGs) based on nominal *p*-value and false discovery rate (FDR). Furthermore, the subnetwork modules were constructed and the obtained DEGs were analyzed for the biological processes, molecular components, KEGG pathways and cellular component analysis. Finally, we mapped out hub genes from the differentially expressed genes network that could act as possible drugs targets.

## Methods

### Microarray data

**Fig. 1:**
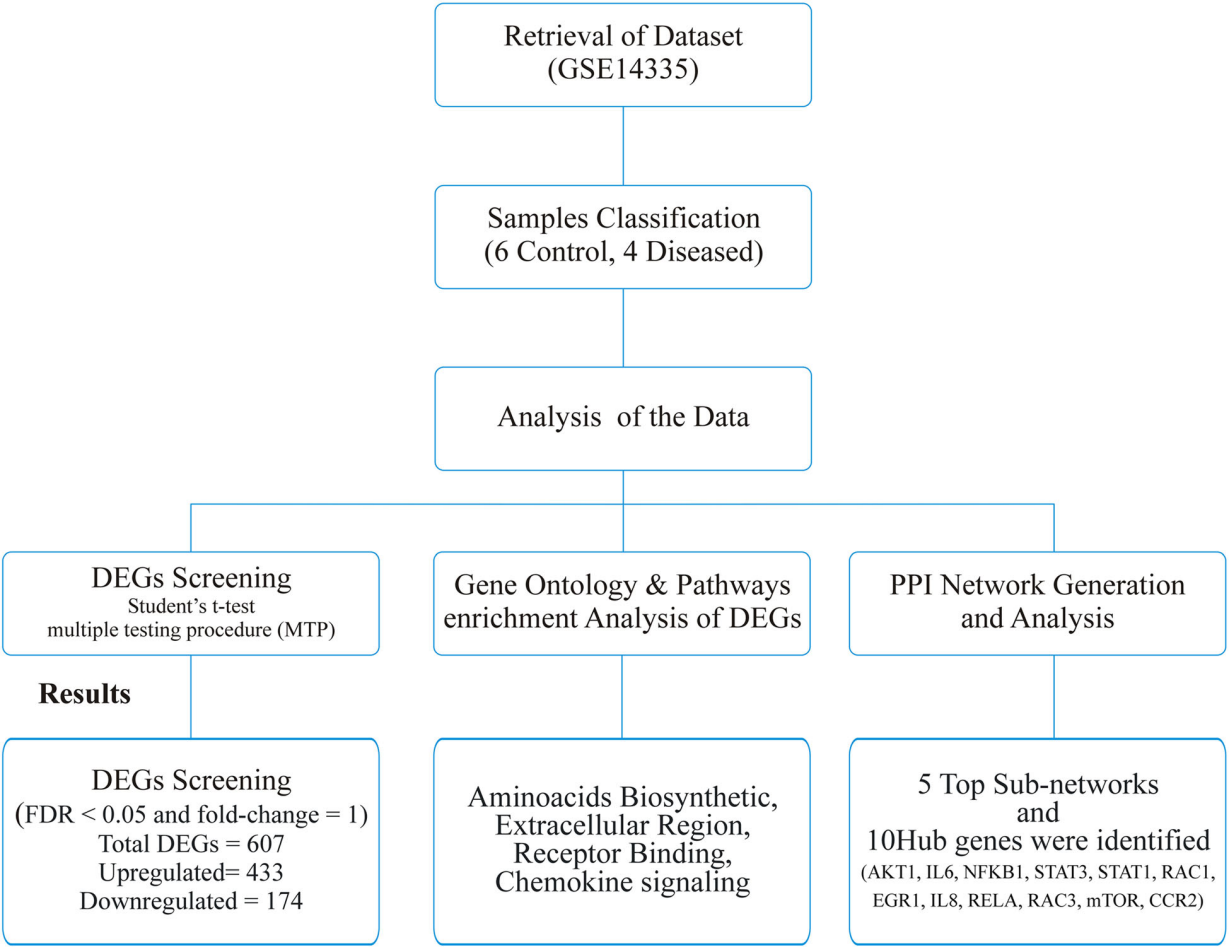
Schematic diagram for the gene expression dataset and protein-protein interaction network analysis. The flow is also showing the major results obtained from these analyses

Differential analysis of one channel microarray data (Accession No:GSE14335) [27] based on the GPL570 Affymetrix Human Genome U133A plus 2.0 Array platform was retrieved from NCBI GEO (http://www.ncbi.nlm.nih.gov/geo/) [28]. The dataset submitted by *Avondo* et al. *2009* consists of ten samples including four DBA and six control samples. Pre-processing and differential analysis of the dataset was carried out to conclude the final results. In the pre-processing, a collectively phenotypic and the samples information stored in .cel files were combined to precisely approach the differential expression step. The expression intensity values were subjected to quantile normalization to remove the noise from the microarray data [29] using an integrated GCRMA [30] package of Rstudio v 3.0.2 [31]. Quantile normalization is perhaps the most widely implemented method for considering microarray data produced by Affymetrix GeneChip platform. Following the normalization, an expression set was built which contain information regarding the assay probes, features, phenotype and the experimental setup. Based on the presence or absence of RPS19 gene mutation, the data was divided into two groups including the control and diseased (Table 1). The methodolgical flow of the work as as defined by (ref [32]) is given in the Fig. 1.

**Table 1:** The dataset divided into two groups based on mutation

No	Group No	Number of Samples	Phenotype
1	Group I	4	RPS19 Mutated
2	Group II	6	Normal

### DEGs screening

The expression set contains the log expression values was used to design a model matrix to accurately adjust the total 54,675 probes according to their phenotypic information. This matrix includes a coefficient for the diseased vs normal, rather than applying another approach to assign a separate coefficient to each condition and then using a contrast to extract the difference. A pertinent statistical test such as Student’s *t*-test [33] is applied to these *normalized* gene expression levels. The resulting *p*-values are adjusted by a multiple testing procedure (MTP) in order to control certain quantity of per-family Type I error, such as family-wise error rate (FWER) [34–36] and false discovery rate (FDR) [37]. Differentially expressed genes are identified based on a pre-specified threshold of adjusted *p*-values. More detailed introduction of statistical methods for detecting differentially expressed genes can be found in [32, 38, 39]. Using Benjamin-Hochberg [40] multiple testing method the FDR < 0.05 with fold-change ≥1 and the adjusted p-value < 0.05 was selected as the threshold for DEGs identification.

### Gene ontology and pathway enrichment analysis of DEGs

Gene ontology (GO) is a useful tool to annotate genes and its products. Attributes of high throughput genomics and transcriptomics data could be obtained through GO analysis [41, 42]. KEGG (http://www.genome.jp/kegg/pathway.html) provides a detail information about gene function and pathways and also link the genomics data with the high order functional information [43]. For the functional analysis DAVID (The Database for Annotation, Visualization and Integrated Discovery) (https://david.ncifcrf.gov/) is an essential online server which can functionally annotate genes with high success [44]. Here we used DAVID to label the mapped DEGs to their functional class, pathways and GO enrichment processes. A *p*-value < 0.05 was defined as significant threshold for the essential annotations.

### Protein-protein interaction (PPI) network generation

PPI network of the total DEGs was retrieved from STRING database [45] and GeneMANIA [46]. Current STRING database contains 9,643,763 proteins and 2031 total organisms while GeneMANIA owned 747 data sets. The interactions were loaded into the Cytoscape v 3.4 [47] and were analysed using different integrated functions.

### Network topological parameters

Numbers of topological parameters are available to analyse and compare the network. Cytoscape is a freely available software which provides an integrated function “NetworkAnalyzer” to analyse the gene/protein network. Here we also used “NetworkAnalyzer” to calculate the parameters for all the constructed networks. The primary parameters which were analysed includes power law of node distribution, distribution of node degree, clustering coefficient, network centralization and density to distinguish the three constructed networks [48].

### Hub genes identification

Cytohubba is a well-known integrated plugin in Cytoscape which analyse the network features and rank the nodes in the network accordingly [49]. Cytohubba uses 11 different methods to analyse the network including the identification of hub genes/nodes in a network. We used Cytohubba to find out the hub genes in our constructed network of total DEGs which could be the possible new drug targets for the treatment of DBA.

### Molecular complex detection analysis (MCODE)

MCODE is an automated algorithm which can be used as an integrated plugin in Cytoscape provides a way to identify highly connected dense subnetwork in a PPI/gene networks [50]. To cluster the subnetworks in the total DEGs we also used MCODE. The interconnected nodes in the subgraphs were identified and selected for further analysis based on number of node. We used *n* > 10 as a parameter for selecting highly interconnected sub-networks.

## Results

### Identification of differentially expressed genes

**Fig. 2:**
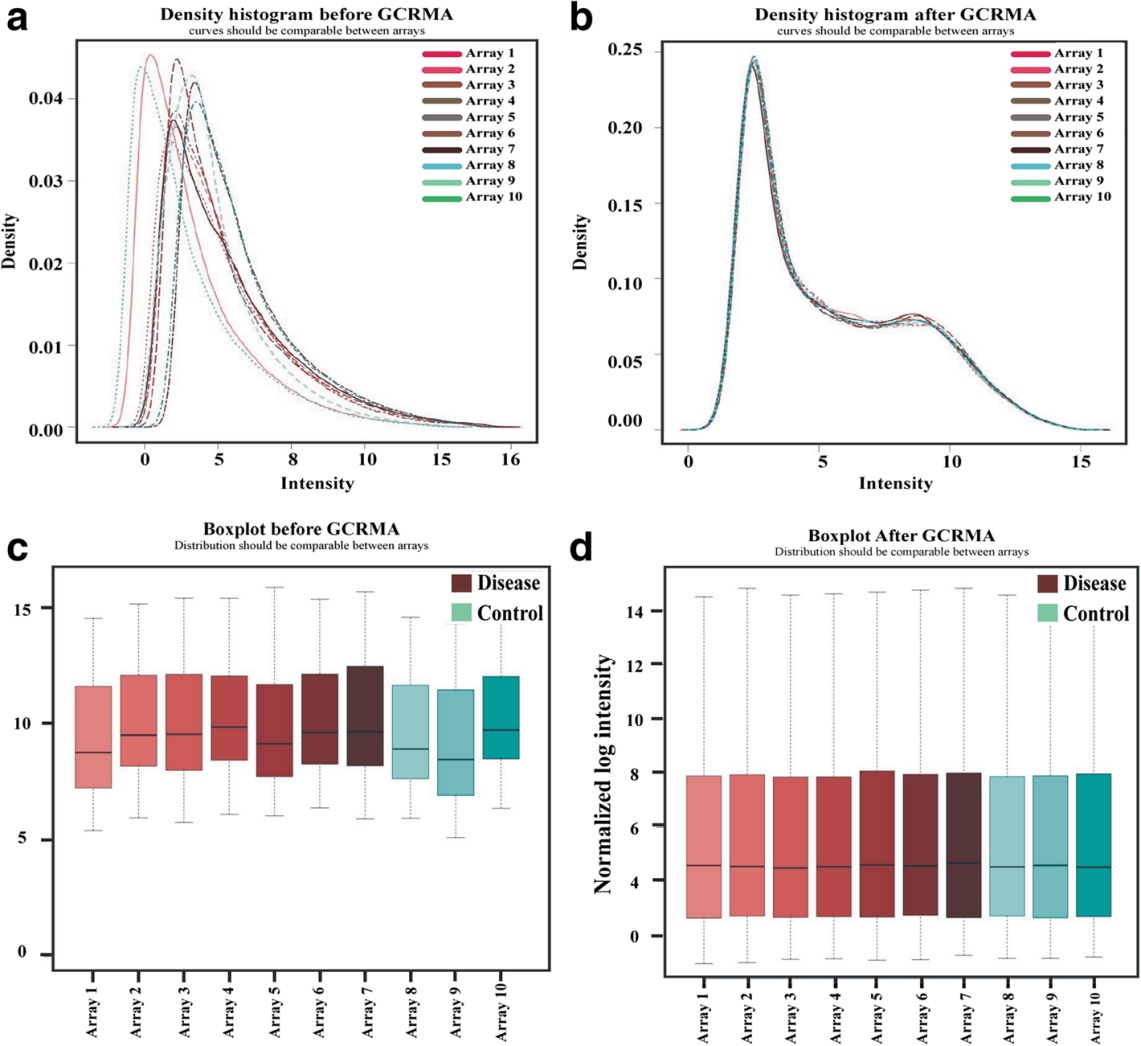
The figure shows the quality of the data before and after normalization. **a** and **b** are the density plot. **a** is before the normalization in which the data is not good enough to be compared while the (**b**) graph is showing the overlapped density plot. **c** and **d** are the normalization plot which is also revealing the quality of the data is comparable

Analysing the network calculated R^2^ = 0.26 which shows that the network is scale-free. The complex biological system is composed of thousands of genes and its products. These genes and products interact randomly and form a complicated network.. Microarray expression analysis may pose many regular variations. To overcome these variations Normalization process is usually carried out. These variations may reveal different expression level then actual which is a major problem in gene expression analysis. Statistical models are proposed to perform normalization. Here, we also used “ArrayQualityMetrics” and “GCRMA” to perform normalization. The density plots and boxplot of the data before and after normalization is shown in the Fig. 2. Based on the defined criteria (FDR < 0.05 and fold-change ≥1) we compared the two types of samples in the dataset that result a total of 607 differentially expressed genes (Additional file 1: Table S1) using R Bioconductor. Among the total DEGs 433 were upregulated while the rest 174 were downregulated. Using this criteria we identified some novel DEGs with important functions.

### GO term enrichment analysis of the up and downregulated DEGs

**Fig. 3:**
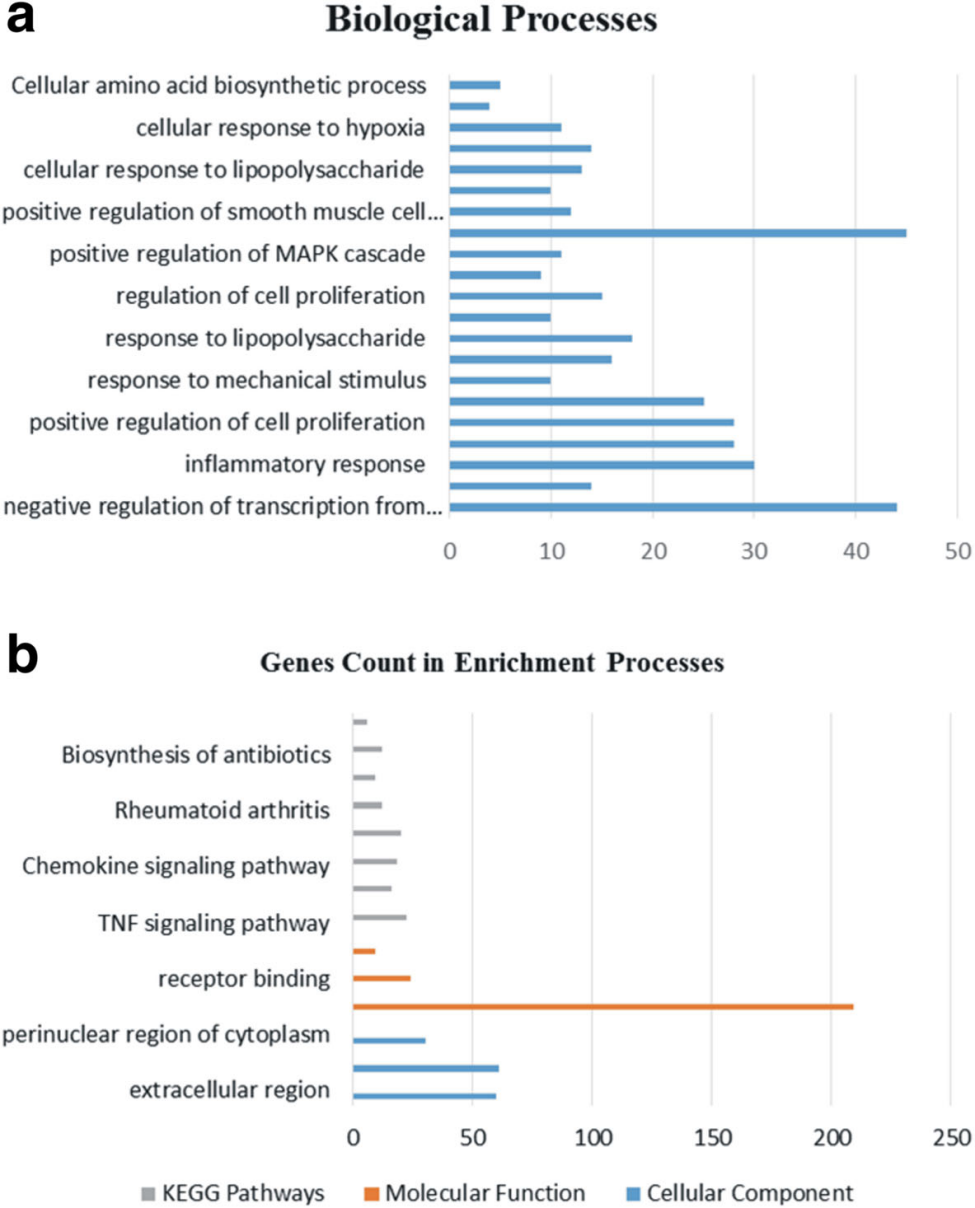
The figure shows the number of genes enriched in different biological processes, cellular components, molecular functions and the KEGG pathways they are involved in. **a** is showing the biological processes only while (**b**) is showing the cellular components, molecular functions and KEGG pathways along with the number of genes involved in it

Functional exploration of functional genomics data can be obtained by utilizing GO analysis process. Here, we also utilized a well-known functional annotation database DAVID to understand the GO processes of identified DEGs. For enrichment analysis of DEGs we selected GO biological processes, GO Molecular function, cellular component and KEGG pathways analysis. In biological processes negative regulation of transcription from RNA polymerase II promoter, chemo taxis, inflammatory response, immune response, positive regulation of cell proliferation, negative regulation of cell proliferation, response to mechanical stimulus, positive regulation of cell migration, response to lipopolysaccharide, response to cytokine, regulation of cell proliferation, positive regulation of tyrosine phosphorylation of Stat3 protein, positive regulation of MAPK cascade, positive regulation of transcription from RNA polymerase II promoter, positive regulation of smooth muscle cell proliferation, chemokine-mediated signaling pathway, cellular response to lipopolysaccharide, cellular response to tumor necrosis factor, cellular response to hypoxia from upregulated while downregulated genes were found to be involved in L-serine biosynthetic process and cellular amino acid biosynthetic process. In molecular function protein binding, receptor binding and chemokine activity were only observed in upregulated genes. Upregulated genes were enriched in TNF signaling pathway, osteoclast differentiation, chemokine signaling pathway, cytokine-cytokine receptor interaction, rheumatoid arthritis, while the only three KEGG pathways found in the downregulated genes were biosynthesis of amino acids, biosynthesis of antibiotics and glycine, serine and threonine metabolism. A detail of these processes including the *p*-value and FDR (0.05) are given in the Table 2 and Fig. 3.

**Table 2:** Gene Ontology/functional enrichment analysis of up and downregulated DEGs associated with DBA patients. The FDR and *p*-value cut-off criteria was set > 0.05. The table is enriched with GO biological processes, Molecular Functions and KEGG pathways

Category	Term	Count	*p*-value	False discovery rate
GO-BP:0000122	negative regulation of transcription from RNA polymerase II promoter	44	6.19E-11	1.10E-07
GO-BP:0006935	chemo taxis	14	7.72E-07	0.001365
GO-BP:0006954	inflammatory response	30	3.82E-10	6.77E-07
GO-BP:0006955	immune response	28	6.64E-08	1.17E-04
GO-BP:0008284	positive regulation of cell proliferation	28	5.10E-07	9.02E-04
GO-BP:0008285	negative regulation of cell proliferation	25	1.01E-06	0.001791
GO-BP:0009612	response to mechanical stimulus	10	1.97E-06	0.003493
GO-BP:0030335	positive regulation of cell migration	16	3.39E-06	0.005999
GO-BP:0032496	response to lipopolysaccharide	18	2.31E-08	4.10E-05
GO-BP:0034097	response to cytokine	10	6.52E-07	0.001153
GO-BP:0042127	regulation of cell proliferation	15	1.68E-05	0.029732
GO-BP:0042517	positive regulation of tyrosine phosphorylation of Stat3 protein	9	5.71E-07	0.001010
GO-BP:0043410	positive regulation of MAPK cascade	11	3.88E-06	0.006861
GO-BP:0045944	positive regulation of transcription from RNA polymerase II promoter	45	2.14E-07	3.78E-04
GO-BP:0048661	positive regulation of smooth muscle cell proliferation	12	1.95E-08	3.45E-05
GO-BP:0070098	chemokine-mediated signaling pathway	10	9.53E-06	0.016859
GO-BP:0071222	cellular response to lipopolysaccharide	13	2.14E-06	0.003778
GO-BP:0071356	cellular response to tumor necrosis factor	14	2.30E-07	4.07E-04
GO-BP:0071456	cellular response to hypoxia	11	1.80E-05	0.031815
GO-BP:0006564	L-Serine biosynthetic Process	4	1.35E-06	0.002131
GO-BP:0008652	Cellular amino acid biosynthetic process	5	3.07E-05	0.048645
GO-CC:0005576	extracellular region	60	1.27E-07	1.71E-04
GO-CC:0005615	extracellular space	61	6.12E-11	8.24E-08
GO-CC:0048471	perinuclear region of cytoplasm	30	3.33E-06	0.004483
GO-MF:0005515	protein binding	209	1.90E-07	2.80E-04
GO-MF:0005102	receptor binding	24	2.07E-07	3.05E-04
GO-MF:0008009	chemokine activity	9	3.05E-06	0.004492
KEGG_Pathway	TNF signaling pathway	22	7.91E-13	1.01E-09
KEGG_Pathway	Osteoclast differentiation	16	3.16E-06	0.004022
KEGG_Pathway	Chemokine signaling pathway	18	1.52E-05	0.019366
KEGG_Pathway	Cytokine-cytokine receptor interaction	20	2.02E-05	0.025631
KEGG_Pathway	Rheumatoid arthritis	12	2.97E-05	0.037766
KEGG_Pathway	Biosynthesis of amino acids	9	3.22E-07	0.000387
KEGG_Pathway	Biosynthesis of antibiotics	12	3.11E-06	0.003751
KEGG_Pathway	Glycine, serine and threonine metabolism	6	2.62E-05	0.031616

### PPI network analysis

**Fig. 4:**
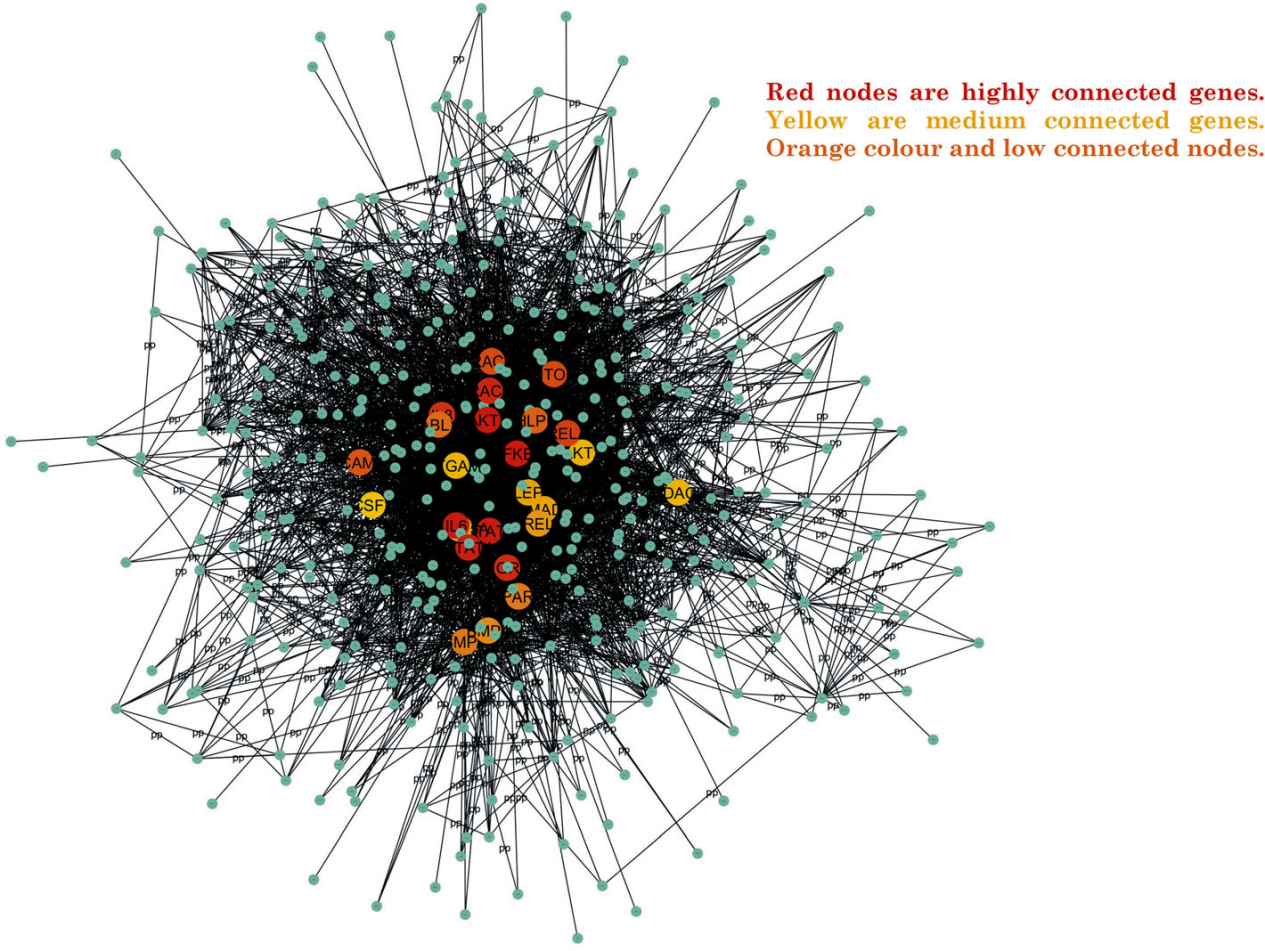
The figure is illustrating the hub genes based on the degree of nodes. Red nodes are highly connected genes, yellow and orange colour are medium and low connected nodes. The Red nodes act more to be hub genes

PPI network is an important approach towards the understanding of biological problems and its elucidation. Nodes and edges are the important combinations to construct a network. The mapped DEGs identified by comparing the data from control and diseased samples were visualized in Cytoscape and hub genes were identified using Cytohubba. Identification of hub genes shown in the Fig. 4 was followed by the identification of highly interconnected dense sub-networks. The k-score was set 2.0, the node score cut off was set 0.2 while the maximum depth for seed node was set 100 for efficiency. MCODE was parameterized by using node degree > 10 which identified a total of 12 modules. Among the 12 modules only 5 were found to be under the defined parameters (nodes > 10 & node score > 2.0). A total of 157proteins and 731 interactions were observed in these sub-network modules. Initial confirmation of the hub genes was carried out by comparing genes from the sub-networks with the identified hub genes, which revealed consistency in the results and confirm the existence of almost all the hub genes in the subnetworks. Final functional annotation of sub-networks and hub genes was carried out to confirm the reliability of our initial results.

**Fig. 5:**
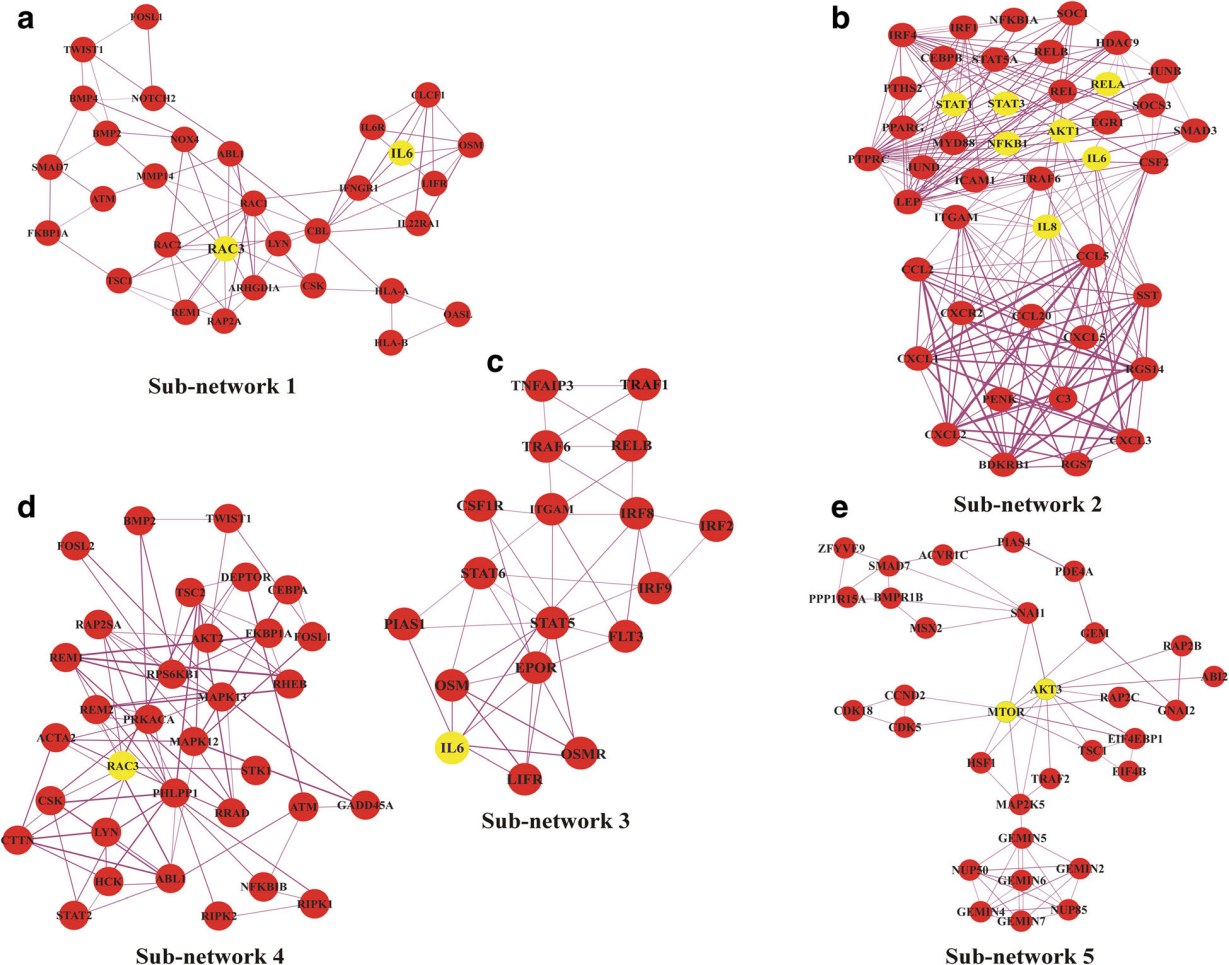
MCODE predicted subnetwork from the protein network of identified DEGs. Yellow colour nodes are hub genes in these subnetworks. While the red are the interconnected genes in the sub-network. (**a**) sub-network 1, (**b**) sub-network 2, (**c**) sub-network 3, (**d**) sub-network 4 and (**e**) sub-network 5

The five different subnetworks generated were as shown in the Fig. 5 and their properties including score, number of proteins and interaction are given in the Table 3. The enrichment analysis of these sub-networks also confirm the validity and they were found to be involved in immune response, inflammatory responses, response to cytokines, chemotaxis, positive and negative regulation of RNA polymerase II transcription activities.

**Table 3:** Statistics for top 5 sub-networks identified in the PPI network constructed from DEGs by MCODE algorithm using Cytoscape

Sub-Network	Score	Proteins	Interaction
1.	8.18	33	131
2.	21.67	44	466
3.	5.67	18	48
4.	3.4	30	60
5.	4.45	32	69

## Discussion

Many different computational approaches such as Single Nucleotide polymorphism (SNPs) analysis, Genome wide association studies (GWAS), diseasome, pathway biomarkers, network module biomarkers and especially gene expression microarray analysis are available to analyze different genomics data and access significant information regarding the disease condition, ranging from diagnosis to treatment [51]. All these approaches can be used to access the available diverse data from different levels including genomics, proteomics, transcriptomics, metagenomics, epigenomics, and metabolomics to frequently aid the prediction and development of both predictive and prognostic biomarkers. PPI network analysis has been widely utilized to support the process of understanding the mechanism of different diseases, identifying drug targets and metabolic processes. The systemic interactions of different proteins in different biological process ranging from normal to disease phenotypes are playing significant role. Analysing microarray gene expression dataset and the identification of differentially expressed genes in a diseased condition compared to the normal, provides a way of targeting different nodes for the discovery of novel drug candidates. It was reported that biomarkers related to a disease through molecular network interactions are more accurate and vigorous. Recently a new method for the identification of miRNAs related to cancer without the prior knowledge of miRNA expression profiling. This method also explains how such miRNA aid the development and progression of cancer [52]. Here, we used microarray gene expression dataset submitted to GEO under accession number GSE14335. Different statistical analysis were carried out such as student-*t* test, Pearson correlation test and Benjamin-Hochberg multiple testing method (FDR < 0.05 with fold-change ≥1) and the adjusted *p*-value (< 0.05) was selected as the threshold for DEGs identification, which result a total of 607 differentially expressed genes, of which 433 were upregulated while the rest 174 were downregulated. Among the downregulated genes COMP (cartilage oligomeric matrix protein) was found to be to most downregulated gene with the fold change value of − 4.003 followed by COL15A1 (collagen type XV alpha 1 chain) and WFDC1(WAP four-disulfide core domain 1) with the fold change values of − 3.188 and − 3.050 respectively. Of the identified upregulated DEGs, EGFL6 (EGF like domain multiple 6) with fold change 4.27, TNFAIP3 (TNF alpha induced protein 3) and SERPINB2 (serpin family B member 2) with 4.49 and 5.33 fold change respectively. Upon subjection to enrichment analysis these DEGs revealed that they are involved in diverse array of processes. Among them the most enriched GO biological processes were immune response, positive and negative regulation of cell proliferation, positive regulation of transcription from RNA polymerase II promoter, extracellular region, extracellular space and protein binding. However, Positive regulation of cell proliferation, negative regulation of cell proliferation, L-Serine biosynthetic Process, Cellular amino acid biosynthetic process, Biosynthesis of amino acids, Glycine, serine and threonine metabolism reported to be associated with DBA [27]. Mapping of DEGs on Cytoscape top 10 hub genes were identified using Cytohubba. For hub genes degree in Cytohubba was set as a parameter. Among the top hub genes AKT1 with highest degree 171, IL6 and NFKB1 with 101, STAT3 with 99 and MTOR with 75 degree was found in the total DEGs network. These hub genes were also found in sub-networks shown in the Table 3 which is a way of validating out results.

**Table 4:** Significant GO Biological terms and pathway analysis for top 10 hub genes identified from the whole PPI network of DEGs based degree of node

Gene	Degree	GO Term
AKT1	171	cellular response to lipopolysaccharide, response to cytokine, inflammatory response, negative regulation of transcription from RNA polymerase II promoter, immune response
IL6	101	cellular response to tumor necrosis factor, positive regulation of smooth muscle cell proliferation, negative regulation of cell proliferation
NFKB1	101	positive regulation of macromolecule biosynthetic process, cellular response to tumor necrosis factor, immune response
STAT3	99	positive regulation of cell proliferation, response to cytokine, inflammatory response
STAT1	91	cellular response to tumor necrosis factor, cellular response to lipopolysaccharide, response to cytokine, immune response
RAC1	88	immune response
EGR1	79	cellular response to hypoxia
IL8	77	cellular response to lipopolysaccharide, response to cytokine, inflammatory response, immune response, regulation of cell communication
RELA	76	positive regulation of cell proliferation, response to cytokine, inflammatory response
RAC3	75	positive regulation of cell proliferation
mTOR	75	positive regulation of macromolecule biosynthetic process, inflammatory response, immune response

The enrichment of hub genes given in the Table 4 reports the same category of processes and thus validate our results. RPS family is considered to be mostly involved in the causing of DBA. Many different mutations including missense and non-sense mutation in the RPS19 is reported to be the most common. This mutant condition is characterized by haploinsufficiency [53]. Among the hub genes, AKT1 was reported to be the best target for the treatment of DBA. Hypothesis developed from different studies by *Gazda & Sieff* suggested that restriction of recruitment of polysome to other complexes for translation is regulated by AKT pathway. Not only AKT but also mTOR was reported to be involved in the restriction of forming such complexes which result haploinsufficiency [54]. Gazda et al.*, 2006* in reported that Ribosomal Protein Gene Expression Alters Oncogenic Pathways in DBA [55]. Functional enrichment analysis also reported the AKT1 as the negative regulator of transcription from RNA polymerase II promoter Another study conducted by Payne et al., 2012 and Stipanuk *2007*, reported that treatment of RPS19 and RPS14 defected cells using Zebrafish model, skeletal muscle of rats and human CD34^+^ cells reported increased production of proteins. This condition was justified by as that L-leucine activate mTOR pathway and thus improves anemia in the DBA patients by promoting mRNA translation. On the other hand Isoquinoline-5-sulfonamides, Azepane derivatives, Aminofurazans, heterocyclic 6-5 fused rings compounds, Phenylpyrazole derivatives and Thiophenecarboxamides and derivatives are reported inhibitors reverse the negative role AKT1 in diseases. Clinical trials of L-leucine mediated mTOR pathway activation and treatment of DBA is under process [56, 57]. IL-6 with degree value of 101 in the hub genes could be another possible target for DBA treatment. It has been confirmed while culturing of DBA patient cells in a liquid containing IL-6 media reported decrease proliferation [58]. Furthermore, the analysis reported by previous disseminate interfaces interaction between RPS19 and the proto-oncogenic protein PIM-1. This study explained that the interaction RPS19 and the proto-oncogenic protein is mediated by the cytokines in the intracellular environment. Likewise many cytokines such as IL-6 and IL-8 are among the hub genes reported in this study could be the biomarkers to be probed for the robust treatment against the DBA infirmity [16]. This bioinformatics pipeline proposed a list of potential cellular proteins which are identified as hub genes and could be the targets for the treatment of DBA.

## Conclusion

In this study computational systems biology approach was utilized to effectively find DEGs that might lead to DBA condition. A total of 607 DEGs were identified, of which many new upregulated were identified in the DBA samples. Overall, the current findings speculate a potential association of these DEGs with DBA using in silico gene expression patterns and network topological analysis. Among the total identified DEGs EGFL6, TNFAIP3 and SERPINB2 were top upregulated which might implicate in the pathogenesis of DBA through interacting with one another. These findings may provide new insights on the DBA pathogenesis, and lay the foundation for the targeted therapy of the disease. We envision our results from bioinformatics analysis will be validated by experimental work.

## Additional file

Additional file 1: **Table S1.** The identified differently expressed genes are listed in the Table S1. Both upregulated and downregulated genes are given based on the defined criteria. (XLSX 52 kb)

## Abbreviations

CCR2: Chemokine receptor type 2
EGR1: Early Growth Response 1
GEO: Gene Expression Omnibus
GWAS: Genome Wide Association Studies
IL-6: Interleukin-6
IL8: Interleukin 8
mTOR: Mammalian target of rapamycin
NCBI: National Centre for Biotechnology Information
NFKB1: Nuclear factor NF-kappa-B1
RAC1: Ras-related C3 botulinum toxin substrate 1
RAC3: Ras-related C3 botulinum toxin substrate 3
SNP: Single Nucleotide Polymorphism
STAT1: Signal transducer and activator of transcription 1
STAT3: Signal transducer and activator of transcription 3
